# Investigation of antioxidant, antimicrobial, and enzymatic properties of thermophilic cyanobacteria extracts

**DOI:** 10.5114/bta.2024.145253

**Published:** 2024-12-19

**Authors:** Aytan Fataliyeva, Meral Yilmaz Cankilic, Nalan Yilmaz Sariozlu

**Affiliations:** 1 V.Y. Akhundov Scientific Research Medical Preventive Institute, Baku, Azerbaijan; 2 Department of Biology, Eskişehir Technical University (ESTU), Tepebaşı/Eskişehir, Türkiye

**Keywords:** thermophilic cyanobacteria, enzyme activity, antioxidant, antimicrobial

## Abstract

**Background:**

The present study investigated the antioxidant, antimicrobial, and partial enzymatic properties of 52 thermophilic cyanobacteria isolates *in vitro*.

**Materials and methods:**

The DPPH scavenging method was applied to test the antioxidant potential of isolates’ methanol extracts. Agar block diffusion and agar well diffusion methods were used to evaluate the antimicrobial activity and measured in milimeters. The measurement of enzyme activity was carried out by a modification of the agar block method by the growth of the cyanobacteria.

**Results:**

Among the cyanobacterial extracts, strain 37 (0.78 ± 0.055 mg/ml) showed an IC_50_ value close to ascorbic acid (0.22 ± 0.04 mg/ml), indicating that it has a specific antioxidant source. Isolate G13 was shown to have the strongest antimicrobial activity against *Micrococcus luteus* NRRL B-4375 in the agar well diffusion method. In addition, the ability to produce enzymes was determined in isolate G1 (25 ± 5.66 mm), which had the highest cellulase activity at pH 8, and isolate K42 (22 ± 0.71 mm), which had the highest lipase activity at pH 7.

**Conclusion:**

When percent inhibition and IC_50_ values were examined, it was found that cyanobacterial methanol extracts had moderate and low scavenging activity in comparison to the standard antioxidant ascorbic acid. In a study using the agar well diffusion method, the activity of cyanobacterial extracts against test bacteria was observed. In light of the results obtained, it is believed that the isolates exhibited lipase and cellulase (pH 7 and 8) enzyme activity at both pH levels and have potential for industrial use.

## Introduction

Cyanobacteria are prokaryotic microorganisms that, throughout their evolutionary history, have played an important role in nitrogen fixation and the global carbon cycle, and have cellular mechanisms to adapt to environmental changes and rapid population growth (Sukenik et al., 2012; Sánchez-Baracaldo et al., 2022). In the last decade, cyanobacteria have been identified, and their chemical structure and pharmacological activities have been studied extensively (Zahra et al., 2020).

Thermophilic cyanobacteria (blue–green algae) are defined as microorganisms that can thrive at temperatures of 45°C or higher in their optimum growth range and are the primary producers of economically important metabolites (Paerl and Paul, 2012; Gaysina et al., 2019).

Cyanobacteria are known to produce a variety of therapeutically effective bio-compounds that can be obtained from bio-mass or extra-cellularly released into the environment (Khalifa et al., 2021). Metabolites extracted from cyanobacteria have been reported to have important bioactivities such as antibacterial, antiviral, antiinflammatory, antitumor, antimalarial, immunosuppressant, and anti-HIV (human immunodeficiency virus)

(Tabarzad et al., 2020; He et al., 2024). Since cyanobacteria are still largely undiscovered, they offer a significant opportunity for the discovery of new metabolites. The antioxidant compounds derived from cyanobacteria provide a natural alternative to existing synthetic antioxidants (Guerreiro et al., 2020). In general, the studies point to an interest in discovering the antioxidant activity of cyanobacteria, taking into account the richness of compounds such as carotenoids and polyphenols. Furthermore, some researchers (Geethu and Shamina, 2018) have reported that antioxidant contents in cyanobacteria are quite similar and sometimes higher than those found in eukaryotic microalgae, macroalgae, or higher plants (Hossain et al., 2016; Rajishamol et al., 2016). The occurrence of cyanobacteria in a variety of habitats makes them a promising source for research on antimicrobial activity due to their ability to utilize H_2_O and CO_2_ as a low-cost source of inorganic nitrogen and phosphorus and with the help of solar energy. Cyanobacteria have a versatile use as biofabricates because they can synthesize a wide range of enzymes (Markets, 2015). Although less studied than other organisms, new enzymes are discovered every day in the chemical, pharmaceutical, and food industries (Perera et al., 2023; Enzing et al., 2014). New strategies are still needed to optimize the development of cyanobacteria that produce these enzymes on an industrial scale. The production of enzymes of bacterial origin is a common application of industrial biotechnology (Mogharabi and Faramarzi, 2016). The enzymes produced include hydrolytic thermostable enzymes for biofuel production such as amylases, cellulases, proteases, and xylanases (Odenthal et al., 2024; Gifuni et al., 2019). Uncovering cyanobacterial genetic diversity will provide access to new genes, including industrially valuable enzyme-coding genes for future biotechnological discoveries (Srivastava et al., 2018; Willis et al., 2020).

The study investigated the antioxidant and antimicrobial activity of thermophilic cyanobacterium extracts and the enzyme activity of cultures.

### Cyanobacteria samples and culture media

In the study, thermophilic cyanobacteria isolated from a thermal spa area in Kütahya, Türkiye, and preserved in the Microbiology laboratory of Eskisehir Technical University were used. Hundred microliter of BG-11 medium was prepared and sterilized in 250 ml flasks and stock cultures were incubated in this medium for 4–5 weeks at 45°C in an air conditioning cabinet (Climacell) (Stanier et al., 1971; Abelson and Simon, 1988).

### Preparation of extracts

After obtaining cyanobacterial biomass in the BG-11 broth medium, it was centrifugated for 10 min at 10 000 rpm in sterile Falcon tubes, and the obtained pellets were washed twice with sterile distilled water. After the pellets were dried at 45°C, they were crushed in mortar to become powder and extracted at 4°C with 100% methanol for 24 h. Then sonication (Sonics Materials) to break down the cell walls at 40 kHz for 5 min. The supernatant was separated by centrifugation at 10 000 × g for 10 min and successively filtered through a syringe filter (Minisart, NY 0.45 μm). After the soluble fraction is dried in the Rotary evaporate evaporator at a controlled temperature, the powder extract is obtained.

### Evaluation of antioxidant properties

#### DPPH free radical scavenging activity

The scavenging effect of cyanobacterial extracts on DPPH-(2,2-diphenyl-1-picrylhydrazyl) free radical was assayed according to Mensor et al. (2001). The assay is based on the ability of a solution to donate protons or electrons, i.e., the ability of the DPPH solution to lighten from purple to pale yellow. Samples prepared at a concentration of 10 mg/ml were diluted 1 : 1 with methanol in tubes and five different concentrations of solution were prepared. Then 3 ml of 0.06 mM DPPH-solution was added to 1 ml of methanolic cyanobacteria extract and mixed in the tube. The reaction mixtures were kept in the dark for 30 min. In the DPPH^•^ method, the prepared 0.06 mM DPPH^•^· solution, prepared fresh every day and wrapped in aluminum foil, should be stored in a dark environment at +4°C. The color change in the reaction mixture was read as absorbance at 520 nm using a UV/VIS Spectrophotometer. Methanol was used as a blank. An ascorbic acid solution of 1 mg/ml was prepared as a standard solution. Percent inhibition value for the sample and synthetic antioxidant was calculated according to the formula below.


$$\text{Inhibition }[\%]\;=\;(A_{0}-A_{1}/A_{0})\times 100\%$$ (1)

*A*_0_ is the absorbance of the control, *A*_1_ is the absorbance of the test sample. Antioxidant activity was expressed as the concentration of the extract that scavenged 50% of DPPHradicals (IC_50_) calculated by nonlinear regression analysis.

### Determination of the antimicrobial activity of cyanobacteria

#### Determination of antimicrobial activity by agar block diffusion method

Cyanobacteria isolates were cultured on BG-11 agar medium and grown in an acclimatization chamber at 45°C for 20 days for antimicrobial activity studies. Cyanobacteria-containing agar blocks with a diameter of 8 mm were removed from each Petri dish containing these long-grown isolates. To determine whether these agar blocks contain the cellular, bioactive extracellular metabolites synthesized by cyanobacteria, these agar blocks were compared with test microorganisms and evaluated for activity. As test microorganisms *Bacillus cereus* ATCC 10876, *Escherichia coli* ATCC 25922, *Enterococcus faecalis* ATCC 51299, *Klebsiella pneumoniae* ATCC 700603, *Micrococcus luteus* NRRL B-4375, *Staphylococcus aureus* ATCC 6538, *Salmonella typhimurium* ATCC 14028, *Bacillus subtilis*, *Enterobacter aerogenes* ATCC 1304, *Listeria monocytogenes* ATCC 19111, *Proteus vulgaris* NRRL B-12*3*, *Streptococcus faecalis* NRRL B-14617, *Yersinia enterocolitica* Y53 bacteria, *Candida albicans* ATCC 90028, *Candida glabrata* ATCC 90030, *Candida parapsilosis* ATCC 22019 and *Candida krusei* ATCC 6258 yeasts were used. Cyanobacteria agar blocks were placed on bacterial and yeast cultures that had been inoculated with sterile swabs onto MHA and SDA medium, respectively. The cultures were then incubated for 24 h at 37°C. Agar blocks of medium without cyanobacteria cultures were used as negative control and coloramphenicol for bacteria and ketoconazole for yeasts were used as positive control. Inhibition zones formed around the agar blocks at the end of incubation were measured (Balouiri et al., 2016).

#### Determination of antimicrobial activity by agar well diffusion method

The antimicrobial activity of intracellular methanol extracts of cyanobacteria has been determined according to the Clinical and Laboratory Standards Institute (CLSI, 2017) method (Balouiri et al., 2016). Hundred microliter of microorganism cultures were inoculated on MHA medium for bacteria and SDA medium for yeasts by smear inoculation method. Then, wells were formed on the Petri dishes with the help of a sterile agar drill with a diameter of 8 mm. Fifty microliter of cyanobacteria extract dissolved in 20% DMSO at 5 mg/ml was added to the wells of each Petri dish using a micropipette and the Petri dishes were incubated at 37°C for 24 h. The extracts thought to contain antimicrobial components were assessed based on whether or not they created an inhibition zone surrounding the disks. The zones of inhibition were recorded in mm. As a negative control, 20% DMSO was used to dissolve the extracts and as a positive control, coloramphenicol for bacteria and ketoconazole for yeasts were used.

### Determination of enzyme activities of thermophilic cyanobacteria

#### Determination of protease enzyme activity

The proteolytic enzyme activity of thermophilic cyanobacteria was determined using 6 g/l skim milk or gelatin, 16 g/l agar, and BG11 nutrient (pH 7 or pH 8). Agar blocks previously extracted from cyanobacteria cultures with a diameter of 8 mm were placed on a medium containing skim milk or gelatin and left to incubate at 45°C for 4–5 days with 12 h of light and 12 h of darkness. At the end of incubation, inhibition zones were measured by determining transparent areas around the agar blocks (de Veras et al., 2018). Blank agar blocks without cyanobacteria were used as a negative control.

#### Determination of lipase enzyme activity

In our research, the lipolytic enzyme activity of thermophilic cyanobacteria has been tested with 6 ml of tributyrin, 16 g/l agar, and BG11 nutrients (pH 7 or pH 8). 8 mm diameter agar blocks extracted from cyanobacterial cultures are placed on solid tributyrin feed and incubated for 4–5 days at 45°C for 12 h of light–12 h of darkness. Agar blocks without cyanobacteria were used as negative control. Lipase enzyme activity was determined by measuring the hydrolysis site obtained (de Veras et al., 2018).

#### Determination of amylase enzyme activity

For the determination of amylase enzyme activity, 10 g/l starch, 16 g/l agar, and BG11 medium (pH 7 or pH 8) were used. In the determination of amylase enzyme activity, agar blocks extracted from cyanobacteria cultures were placed on the starchy medium using a sterile agar punch with a diameter of 8 mm and incubated at 45°C for 4–5 days with 12 h of light and 12 h of darkness. At the end of incubation, Lugol’s solution was added to the Petri dish, and staining was performed for 15 min if starch was present, the ground was stained dark blue. Hydrolysis of starch resulted in the formation of a clear transparent zone around the amylase-producing disks (Aşule, 2019).

#### Determination of cellulase enzyme activity

Enzyme activity was determined using a medium (pH 7 or pH 8) containing 1 g/l carboxymethylcellulose (CMC), 16 g/l agar and BG11 (de Veras et al., 2018). Eight millimeter diameter agar blocks containing cyanobacteria were sown on medium containing carboxymethylcellulose (CMC) and the samples were incubated at 45°C for 4–5 days with 12 h of light and 12 ho of darkness. After incubation, 0.1% Congo red solution was poured into the Petri dishes, and staining was performed for 15 min. After staining, 1 M NaCl solution was added to the medium and incubated for 15 min to remove the remaining dye. Activity was considered positive if a yellow hydrolysis zone was formed (Voget et al., 2006).

### Statistical analysis

The results obtained were done in 2 or 3 replicates and the data are shown as mean ± standard deviation (SD).

## Results

### DPPH free radical scavenging test results

**Fig. 1:**
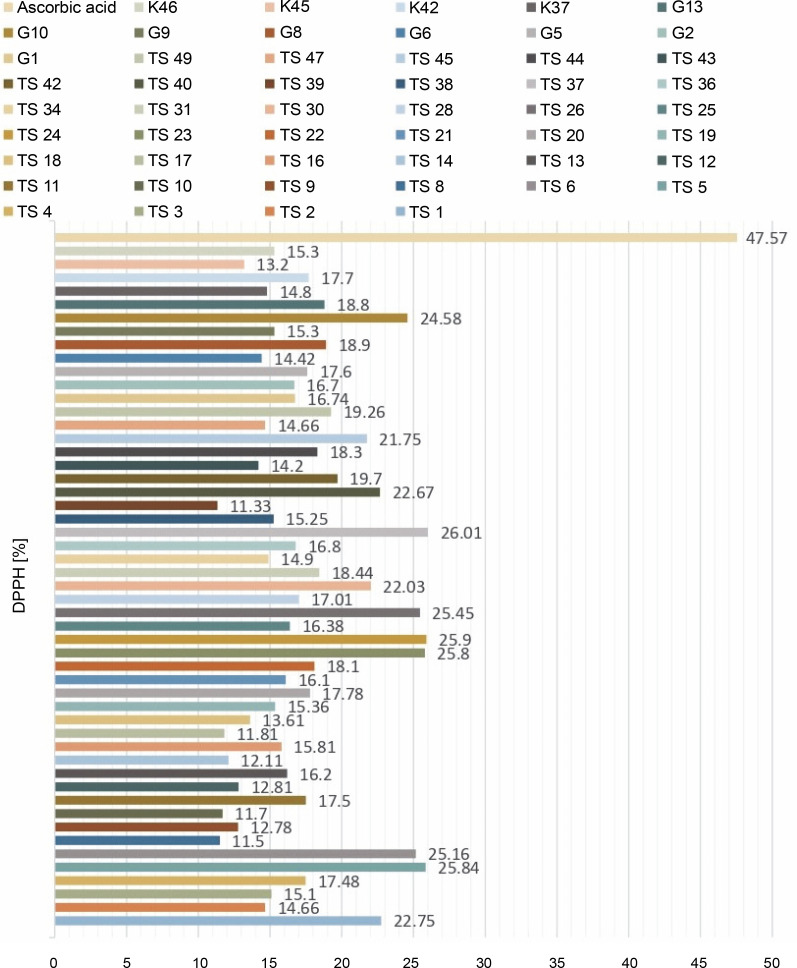
DPPH-quenching graph of methanolic extracts (0.2 mg/ml) of cyanobacteria isolates

**Fig. 2:**
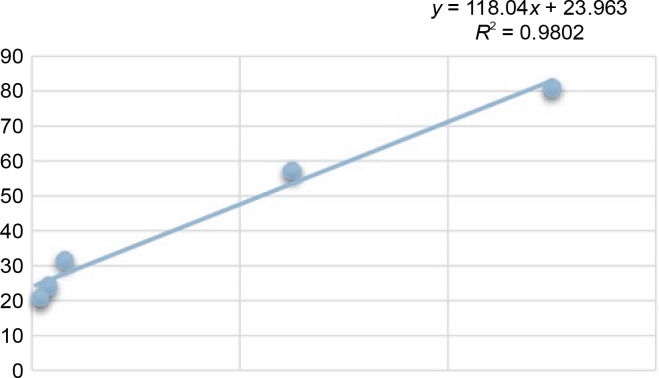
Calibration graph of ascorbic acid

For the determination of free radical scavenging activity, firstly, percent of DPPH-quenching values were calculated for the sample and synthetic antioxidant. The graph of DPPH-quenching of methanolic extracts (0.2 mg/ml) of each cyanobacteria isolates is shown in Figure 1. Ascorbic acid was used as a synthetic antioxidant for comparison (Fig. 2). The higher the percentage of inhibition, the higher the antioxidant effect. When the values were examined, the synthetic antioxidant ascorbic acid (47.57%) showed a high inhibition value, but in the samples studied, the radicalization effect of isolate No. 37 was higher compared to other isolates (26.01%) has been determined. Sample 39 showed low DPPH-scavenging activity (11.33%) compared to the other isolates. According to Figure 1, it was understood that the percent of inhibition values of thermophilic cyanobacteria isolates showed DPPH-scavenging activity between 25.9 and 11.50%.

**Fig. 3:**
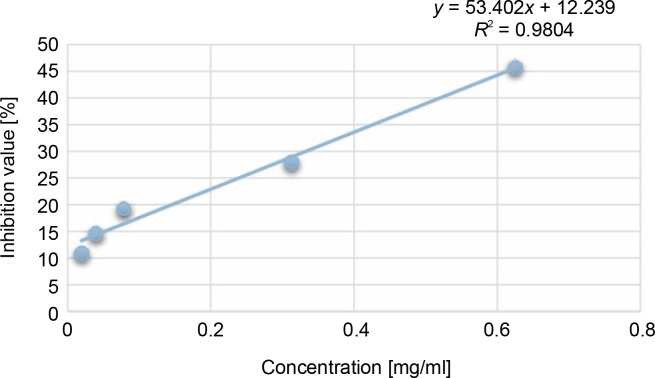
Calibration chart of cyanobacteria extract

**Fig. 4:**
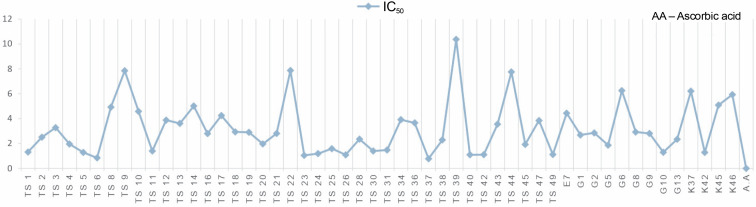
IC_50_ values of methanolic extracts of cyanobacteria isolates

Another indicator of the DPPH radical scavenging effect is IC_50_ values. For each extract concentration (10 mg/ml), the DPPH-quenching graph of the measurements taken in three replicates was drawn and IC_50_ values (mg/ml) were calculated (Fig. 3). The IC_50_ values (mg/ml) of DPPH-quenching of methanolic extracts obtained from thermophilic cyanobacteria isolates are shown in Figure 4. When the graph in Figure 2 is analyzed, the IC_50_ value of DPPHquenching depending on the concentration of AA was calculated as 0.22 ± 0.04 mg/ml.

The isolates showed low DPPH-scavenging activity compared to the synthetic antioxidant ascorbic acid. Among the isolates, isolate 39 (10.37 ± 1.191 mg/ml) exhibited the lowest scavenging activity.

### Determination of antimicrobial activity of thermophilic cyanobacteria

#### Determination of antimicrobial activity by agar block diffusion method

Based on the prediction that extracellular metabolites, if they have antimicrobial properties, will diffuse into the growth medium BG-11 agar, it was applied to visualize the effect on the test microorganisms as zone diameter. Thermophilic cyanobacteria isolates did not show any activity against the test microorganisms used. Since this experiment was designed to investigate the effect of possible extracellular metabolites, it was as sumed that either there were no bioactive metabolites or, if there were bioactive metabolites, the amount secreted into the agar medium in the Petri dish was not high enough to show activity on the test microorganisms. Since the method applied is only a screening and does not provide quantitative data, our experimental results are interpreted only in this way.

#### Determination of antimicrobial activity by agar well diffusion method

Methanolic extracts belonging to the isolates were used to determine the antimicrobial activity of the intracellular metabolites of 52 thermophilic cyanobacterial isolates. As a result of the experiment, no activity was observed against the bacteria *Streptococcus faecalis* NRRL B-14617*, Escherichia coli* ATCC 25922*, Yersinia enterocolitica* Y53*, Salmonella typhimurium* ATCC 14028*, Enterobacter faecalis, Listeria monocytogenes* ATCC 19111.

Inhibition zones were formed around the wells if there was activity, and no inhibition zone was formed if there was no activity. No antimicrobial activity was observed in %DMSO used as a negative control as expected. Cyanobacteria isolates showed very low activity compared to Chloramphenicol used as a positive control.

As shown in Table 1, 24 cyanobacteria isolates showed activity against *Staphylococcus aureus*, an important pathogen. The highest activity was obtained with isolates G1 (12.5 ± 2.12 mm) and G2 (12.5 ± 2.12 mm) and the lowest activity was obtained with isolate 37 (8.75 ± 0.35 mm). Eighteen of the isolates showed activity against *Bacillus subtilis* bacteria. Isolates G1 (12 ± 0.0 mm), G5 (12 ± 0.0 mm), and K42 (12 ± 0.0 mm) showed the highest activity, and isolate 5 (9 ± 0.0 mm) showed the lowest activity. Twelve cyanobacteria isolates exhibited activity against *Enterobacter aerogenes* bacteria, with isolates G2 (11.5 ± 2.12 mm), G5 (11.5 ± 0.71 mm), K42 (11.5 ± 0.71 mm) having more activity and isolate G13 (8.5 ± 0.71 mm) having the least activity. The *Klebsiella pneumoniae* pathogen was inhibited by 23 out of 52 thermophilic cyanobacteria isolates. The maximum activity was obtained with isolates 18 (12.5 ± 0.71 mm) and G2 (12.5 ± 0.71 mm), while the minimum activity was obtained with cyanobacteria isolates 5 (8.75 ± 0.35 mm) and 25 (8.75 ± 0.35 mm). Thirty cyanobacteria isolates also showed activity against *Proteus vulgaris* bacteria. Isolates 2 (11.5 ± 0.71 mm) and 18 (11.5 ± 1.41 mm) showed the highest activity, while isolates 9 (8.75 ± 0.35 mm), 11 (8.75 ± 0.35 mm) and 30 (8.75 ± 0.35 mm) showed the lowest activity. Eleven cyanobacteria isolates showed activity against *Bacillus cereus* bacteria, of which isolate 21 (10.5 ± 0.71 mm) had the highest antimicrobial activity and isolates 2 (8.75 ± 0.35 mm) and 39 (8.75 ± 0.35 mm) had the lowest antimicrobial activity. Seven cyanobacteria isolates were found to be active against *Micrococcus luteus* bacteria, with isolate K37 (9 ± 1.41 mm) showing the least activity and isolate G13 (14 ± 1.41 mm) showing the most activity.

**Table 1:** The values in mm of the zones obtained by agar well diffusion method of thermophilic cyanobacteria isolates

Inhibition zone diameters against test bacteria [mm]							
Isolate number	Staphylococcus aureus	Bacillus subtilis	Enterobacter aerogenes	Klebsiella pneumoniae	Proteus vulgaris	Bacillus cereus	Micrococcus luteus
1	–	–	–	9 ± 1.41	10 ± 0.71	–	–
2	12 ± 0.0	10.5 ± 0.7	9.5 ± 0.7	9.75 ± 0.35	11.5 ± 0.7	8.75 ± 0.3	–
4	12 ± 0.0	–	–	9 ± 0.71	9 ± 1.41	–	–
5	9.5 ± 0.71	9 ± 0.0	–	8.75 ± 0.35	9 ± 0.71	–	–
8	–	–	–	9 ± 0.71	11 ± 0.71	–	–
9	–	–	–	–	8.75 ± 0.35	–	–
10	–	–	–	–	9 ± 0.71	–	–
11	11 ± 1.41	10.5 ± 0.71	–	9.25 ± 0.35	8.75 ± 0.35	–	–
12	10.5 ± 2.12	–	–	8.8 ± 0.28	9.5 ± 0.71	–	–
13	9 ± 0.0	–	–	9 ± 0.71	10 ± 1.41	–	–
16	–	9.5 ± 0.71	–	–	–	–	–
17	9.75 ± 0.35	10 ± 1.41	–	12 ± 0.0	10.5 ± 0.71	–	–
18	11 ± 1.41	11.5 ± 0.71	–	12.5 ± 0.71	11.5 ± 1.41	–	–
19	9.5 ± 0.71	10 ± 1.41	–	9 ± 0.71	9.5 ± 1.41	–	–
20	–	11.5 ± 0.71	–	11 ± 0.71	11 ± 0.71	9 ± 1.41	10.7 ± 0.3
21	11.5 ± 0.71	11.5 ± 0.71	–	10.75 ± 0.35	10 ± 0.71	10.5 ± 0.71	–
25	10.5 ± 0.71	9.5 ± 0.71	–	8.75 ± 0.35	10.75 ± 0.35	–	–
26	–	–	–	9 ± 0.71	–	–	–
30	–	–	–	9 ± 1.41	10 ± 0.71	–	–
34	10 ± 1.41	–	9.8 ± 0.28	10.7 ± 0.42	9.8 ± 0.28	9 ± 0.14	–
37	8.75 ± 0.35	–	–	–	8.75 ± 0.35	–	–
38	11 ± 0.0	9.5 ± 0.71	9.7 ± 0.35	–	10 ± 0.21	–	–
39	11 ± 0.0	–	–	–	9 ± 0.0	8.75 ± 0.35	–
45	10 ± 0.71	–	10 ± 0.0	–	10 ± 0.35	–	–
E7	10 ± 0.0	10.5 ± 0.71	–	–	9 ± 0.14	–	–
G1	12.5 ± 0.71	12 ± 0.0	11 ± 1.41	11 ± 0.71	10.75 ± 0.35	9.5 ± 0.71	10 ± 0.0
G2	12.5 ± 2.12	11 ± 0.0	11.5 ± 2.12	12.5 ± 0.71	10.5 ± 0.71	9.5 ± 0.71	11 ± 0.0
G5	13 ± 0.0	12 ± 0.0	11.5 ± 0.71	12 ± 0.0	10 ± 0.0	10 ± 1.41	12 ± 1.41
G10	12 ± 1.41	–	9.5 ± 2.12	–	9 ± 0.42	9 ± 0.71	–
G13	–	10 ± 1.41	8.5 ± 0.71	–	–	–	14 ± 1.41
K37	10 ± 0.0	–	10 ± 0.0	12 ± 0.0	9.75 ± 0.35	–	9 ± 1.41
K42	–	12 ± 0.0	11.5 ± 0.71	12 ± 0.0	11 ± 1.06	10 ± 1.41	–
K45	9.5 ± 0.71	–	9.5 ± 0.71	10 ± 0.0	–	–	–
K46	11.5 ± 0.71	11 ± 1.41	11 ± 1.41	11.75 ± 0.35	9 ± 0.42	10 ± 1.41	11 ± 1.41
Kloram-Fenikol	30 ± 0.71	29.75 ± 1.41	30 ± 0.71	32 ± 1.41	33 ± 0.0	33 ± 0.71	34.5 ± 0.7

The antimicrobial activity of cyanobacteria isolates against yeasts could not be determined.

Furthermore, 19 cyanobacteria isolates did not show activity against the tested pathogenic microorganisms. From these results, it can be summarized that the thermophilic cyanobacteria isolates obtained have activity in the range of 7–15 mm zone diameter.

### Enzyme activity determination of thermophilic cyanobacteria

#### Results of amylase enzyme activity

As mentioned in the method section, if starch is present in the medium to which Lugol’s solution is added, violet–blue, if starch is hydrolyzed, a whitish zone is formed under the background. For the evaluation of amylase enzyme activity, BG-11 medium was prepared at two different pH (pH 7 and pH 8) containing starch, and the effect of pH was analyzed.

It is seen that thermophilic cyanobacteria did not form any transparent zone in these media and therefore they were evaluated as negative in terms of their ability to produce amylase enzyme.

#### Results of cellulase enzyme activity

**Fig. 5:**
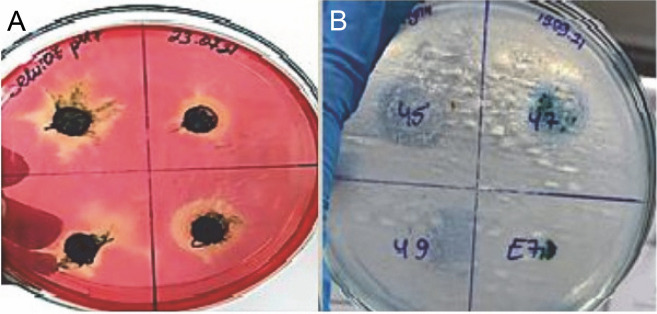
Isolates of thermophilic cyanobacteria: A) cellulase enzyme activity B) lipase enzyme activity

The enzyme activity of cyanobacteria is seen in yellow as shown in Figure 5A, while the unhydrolyzed regions are seen in red.

**Fig. 6:**
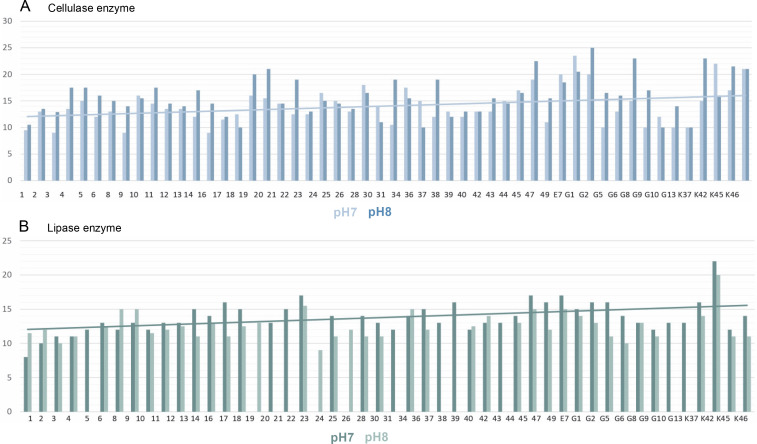
Cyanobacteria pH7, pH8: A) cellulase and B) lipase enzyme activity results (mm)

The pH of the medium was adjusted in two different ways, 7 and 8. The results obtained are summarized in Figure 6A. As can be seen in Figure 5A, if there is cellulase enzyme activity, the red-colored medium turns yellow, and if there is no enzyme activity, there is no change in the red color as there are no hydrolysis zones. The hydrolysis zones of the isolates were recorded as weak (+) for a hydrolysis zone of 1 mm, moderate (++) for a hydrolysis zone of 2–3 mm, and strong (+++) for a hydrolysis zone of more than 3 mm.

According to the results obtained, it was determined that the isolates showed cellulase enzyme activity at both pH values (pH 7 and pH 8). Of 52 thermophilic cyanobacteria isolates, 82.69% had strong cellulase activity (+++), 9.62% had moderate cellulase activity (++) and 7.69% had weak cellulase activity (+) at pH 7. At pH 8, 11.54% of the isolates had moderate cellulase activity (++) and 88.46% had strong cellulase activity (+++). There were no cyanobacteria isolates with weak cellulase activity at pH 8.

Isolates with the highest cellulase activity at pH 7 are identified as E7 (23.5 ± 4.95 mm) and the least cellulose activity is identified by 3 (9 ± 0.71 mm), 9 (9 ± 4.24 mm), 16 (9 ± 5.66 mm). The isolate G1 (25 ± 5.66 mm) with the highest cellulase activity at pH 8 and the isolate with the least cellulose activity as isolate number 1 (10.5 ± 2.12 mm) determined.

#### Results of protease enzyme activity

To determine the protease enzyme activity of thermophilic cyanobacteria, experimental studies were carried out in two different media containing skim milk or gelatin at two different pH values (pH 7 and pH 8). When the isolates were examined, it was found that none of the isolates developed in the substrate-containing medium formed a clear zone. Based on this, it was concluded that these isolates were not capable of proteinase production.

#### Results of lipase enzyme activity

In this study, the lipase enzyme activity of thermophilic cyanobacteria was investigated in BG-11 agar medium containing tributyrin at 2 different pHs (pH 7 and pH 8). The light-colored zones formed around the isolates incubated in the medium after growth indicate enzyme production Figure 5B. Hydrolysis zones of 1 mm were considered as weak (+), 2–3 mm as moderate (++), and more than 3 mm as strong (+++) lipolytic activity. Lipase enzymatic activity was detected at both pH values (pH 7 and pH 8) of the thermophilic cyanobacteria examined. The results obtained are summarized in Figure 6B.

In the medium prepared at pH 7, 78.84% of 52 thermophilic cyanobacteria isolates showed strong lipase activity (+++), 5.77% showed moderate lipase activity (++), 1.92% showed weak lipase activity (+), while 13.46% showed no lipase activity. The isolate with the highest lipase activity at pH 7 was K42 (22 ± 0.71 mm) and the isolate with the lowest lipase activity was isolate 1 (8.5 ± 0.71 mm).

When the activities in the medium prepared at pH 8 were evaluated, 51.92% of 52 thermophilic cyanobacteria isolates had strong (+++), 23.08% had moderate (++), 1.92% had weak lipase activity (+), while 23.08% had no lipase activity. At pH 8, the isolate with the highest lipase activity was K42 (20 ± 3.54 mm) and the isolate with the lowest lipase activity was 24 (9 ± 2.12 mm). Isolates 21, 22, 34, and E7 showed no activity at both pH values.

## Discussion

Physiologically active substances possessing functional properties within the human body are known as bioactive compounds. According to their economic development processes, cyanobacteria – which are known to be environmentally friendly – can be enriched with bioactive compounds as secondary metabolite products with various optimized production controls (Hossain et al., 2016; Jeong et al., 2020). Additionally, it is possible that these microbes can improve health and lower the chance of developing degenerative illnesses (Tabarzad et al., 2020). Studies have shown that compounds such as phenolic compounds, polysaccharides, and carotenoids in cyanobacteria have antioxidant properties, while cyanotoxins, peptides, and fatty acids have antimicrobial effects (Hassan et al., 2022).

In this study, the radical scavenging activities of methanol extracts of 52 cyanobacteria isolates from thermal water sources were investigated by 2,2-diphenyl-1-picrylhydrazyl (DPPH). Antioxidant activity was expressed as the concentration of the extract that scavenged 50% of DPPH-radicals (IC_50_) calculated by non-linear regression analysis. IC_50_ typically represents the amount of a compound required to reduce the free radical concentration by half. Inhibition (%) values and IC_50_ values of cyanobacterial extracts on DPPH-free radical were compared to ascorbic acid as a standard. When we look at the % inhibition and IC_50_ values (Table 1) obtained in our study, it is seen that cyanobacterial methanol extracts showed moderate and low scavenging activity compared to the standard antioxidant ascorbic acid, while ascorbic acid (0.22 ± 0.04 mg/ml) had stronger antioxidant activity than cyanobacterial extracts. According to the IC_50_ values calculated here, isolates 6 (0.85 ± 0.103 mg/ml) and 37 (0.75 ± 0.055 mg/ml) had higher DPPH-scavenging activity than the other samples. According to the percent inhibition values, isolate 37 showed high quenching activity compared to the other isolates (26.01 ± 1.28%) and low quenching activity compared to ascorbic acid (47.57%).

In the cyanobacterial study by Guerreiro et al. (2020), *M. aeruginosa* (LMECYA 127), *Leptolyngbya* sp. (LMECYA 173), *Dolichospermum flos-aquae* (LMECYA 180), *Planktothrix agardhii* (LMECYA 257) and *Planktothrix mougeotii* (LEGE 06224) showed antioxidant activity yields (methanol) ranging from 8.8 to 10.7% by DPPH test. In another study, Senousy et al. (2022) aimed to screen the antioxidant profiles of eight cyanobacterial and two microalgae species using the DPPHfree radical reduction method. *Dunaliella* sp. and *Anabaena* sp. recorded the highest DPPH activity levels with an average of 62.49 ± 0.23% and 61.53 ± 0.23%, respectively. In another study, Şensoy (2019) determined the DPPH radical scavenging activity of carotenoids mixtures obtained from *Geitlerinema sp., Oscillatoria sp*., (A) *Leptolyngbya sp*. and (B) *Leptolyngbya sp*. cyanobacteria. All concentrations of the carotenoid mixture of *Leptolyngbia* (A) are more effective than other species. It was observed that the carotenoid mixture of this species scavenged DPPH radical by 20.33% at a concentration of 800 μg/ml and the DPPH radical scavenging activity increased depending on the concentration. Since cyanobacteria species are not known, a comparison was made with different species studies with the same method. Accordingly, the same or different results are seen.

The study examined the antimicrobial activity of thermophilic cyanobacteria using agar block diffusion and agar well diffusion methods. Antimicrobial activity of cultures against both bacteria and yeasts has not been observed in the agar-blocking method. In the study conducted by agar well diffusion method, cyanobacteria extracts were tested against six of the test bacteria (*Streptococcus faecalis* NRRL B-14617, *Escherichia coli* ATCC 25922, *Yersinia enterocolitica* Y53, *Salmonella typhimurium* ATCC 14028, *Enterobacter faecalis*, *Listeria monocytogenes* ATCC 19111) however, no antimicrobial effect was found against and four yeasts (*Candida albicans* ATCC 90028, *Candida glabrata* ATCC 90030, *Candida 74 parapsilosis* ATCC 22019 and *Candida krusei* ATCC 6258). The highest zone of activity was determined in isolation number G13 (14 ± 1.41 mm) against the bacterium *Micrococcus luteus*. Low, moderate, and high levels of antimicrobial activity against other bacteria have been observed.

Tyagi et al. (2021) measured the antimicrobial activity of 16 different thermophilic cyanobacterial methanol extracts against Gram-positive and Gram-negative pathogens. Based on the size of the bacterial growth inhibition zones, a range of 8.8 ± 0.84 mm to 15.8 ± 0.45 mm was observed against the pathogen *S. aureus ATCC* 25923. In another study by Shaieb et al. 2014, it was determined that cyanobacteria *Nostoc commune* and *A. flavus* species showed significant activity against *K. pneumoniae, E. coli, S. marcescens*, and *Bacillus cereus* in addition to *Micrococcus luteus* pathogen. Shawer et al. (2022) study, the extract from *Limnothrix planktonica* SN4 (MZ504752) among four cyanobacterial extracts showed the highest antibacterial activity against *Salmonella typhi ATCC 15566* and *Bacillus cereus ATSC 12228* with 13.7 ± 1.2 mm and 15.3 ± 1.2 mm inhibition ranges. *Oscillatoria sancta SN2* extract produced maximum zones of inhibition against *P. aeruginosa PTCC 1074* (11.7 ± 0.9 mm), *S. epidermidis* (11.7 ± 1.2 mm) and *Enterococcus faecalis ATCC-29212* (10.3 ± 0.5 mm). However, all extracts showed no antibacterial effect against *E. Coli ATCC-25922*. According to the results of the study, the cyanobacteria examined in this study have important antimicrobial activities and we believe that the biological activities of these substances will give even better results after the isolation, purification, and characterization of some substances from these cyanobacteria species with detailed studies in the future (Gómez-Espinoza et al., 2022).

The method modified to determine enzyme activities has not been studied on cyanobacteria before and there are no reports. The techniques used are literature scans, mostly for enzyme production. Due to the thick cell walls of the cyanobacteria, the breakdown of cyanobacterial cells is inefficient and often laborious (Rasmussen et al., 2016). The study examined the enzyme activity of thermophilic cyanobacterium cultures at two different pH (pH 7 and pH 8) levels. The pH of the growth medium is one of the factors affecting the secretion of enzymes (Paul et al., 2021). Low or high pH values affect the stability of enzymes and can quickly cause denaturation. In general, studies have shown that cyanobacterial enzymes tend to be active at neutral and alkaline pH and give lower results at acidic pH (Ali Shah et al., 2014). According to the results, cyanobacteria were assessed negatively in terms of their ability to produce amylase and protease enzymes. This does not mean that cyanobacteria do not have the activity of amylase and protease enzymes. The method used may not be suitable for the ability of cyanobacteria to produce enzymes. This can be seen in studies carried out for enzyme production purposes. In the study for amylase enzyme, Manoj et al. (2018) investigated amylase enzyme determination in *Rhizoclonium sp. (Dandiganahalli), Spirogyra sp., Klebsormidium sp., Rhizoclonium hieroglyphicum* and *Oedogonium sp*. microalgae, and it was determined that *Rhizoclonium sp. (Dandiganahalli)* showed more amylase enzyme activity. According to Patil and Mahajan (2016), amylase was found to be the least among the basic enzymes found in microalgae, which was explained by associating it with the fact that microalgae are autotrophic.

For the determination of lipase enzyme activity, a tributyrin medium was used at two different pH values. When the pH values were compared, the isolates grown in the medium adjusted to pH 7 gave more efficient results. Only four of the isolates showed no activity. Compared to bacterial, fungal, animal, and plant lipases, lipases found in a few microalgae and the genes encoding lipases were investigated. In this thesis, a lipase was isolated from the photosynthetic cyanobacterium *A. platensis* and characterized for the first time by Demir and Tükel (2010).

A solid medium containing carboxymethylcellulose (CMC) at two different pHs was used for cellulase enzyme activity. When pH values were compared, isolates grown in a medium adjusted to pH 8 gave more efficient results. Although algae and cyanobacteria have genes encoding enzymes, little is known about the production of cellulases (Brasil et al., 2017). The diversity of enzymes found in cyanobacteria suggests their importance as versatile cellular factories. A single set of assay conditions is not optimal for enzymes; the right conditions for one set of enzymes may inhibit others (Spier et al., 2020). For example, methods used to test for specific activities (e.g. addition of reducing agents) or to extract at high temperatures, stringent freeze-thaw cycles will inactivate other enzymes when the conditions are right for several enzymes. More efficient results can be obtained using newer strategies for enzyme activity from cyanobacteria.

## Conclusion

Thermophilic cyanobacteria have been little explored compared to other organisms. As a result, the biological activity of thermophilic cyanobacteria in our study studies have been carried out and positive results have been obtained. The extracts obtained using 52 thermophilic cyanobacteria isolates used in our study were found to have different degrees of antimicrobial effects against bacteria and proved to be used as an aid in the treatment of infections. In addition, more efficient studies using different solvents and advanced extraction methods are required for antioxidant and antimicrobial activity. The chemical components of cyanobacterial extracts should be elucidated and the degree of activity of these components should be investigated by isolating the active substance and adding functional groups. There is also a need to determine the targets and mechanisms of these components. According to the results of the enzyme activities of thermophilic cyanobacteria, amylase, and protease were negative in terms of their ability to produce enzymes, but positive results were obtained in lipase and cellulase enzyme activities. Although most of the genetic resources of cyanobacteria remain unexplored, an increasing number of enzymes with potential applications in the food, pharmaceutical, and chemical industries have been reported. The development of new strategies and methods to promote and optimize microalgae enzyme production, including biomanufacturing, is an important approach for the scale-up and industrialization of potential enzymes. Some of the most important challenges will be to increase the concentration of enzymes and reduce the cost of downstream processing.
